# Behavioral–biological surveillance of emerging infectious diseases among a dynamic cohort in Thailand

**DOI:** 10.1186/s12879-022-07439-7

**Published:** 2022-05-16

**Authors:** Su Yadana, Thaniwan Cheun-Arom, Hongying Li, Emily Hagan, Emma Mendelsohn, Alice Latinne, Stephanie Martinez, Opass Putcharoen, Janthira Homvijitkul, Onarnong Sathaporntheera, Nit Rattanapreeda, Pongtorn Chartpituck, Supalak Yamsakul, Krairoek Sutham, Supharoek Komolsiri, Sonjai Pornphatthananikhom, Sininat Petcharat, Weenassarin Ampoot, Leilani Francisco, Thiravat Hemachudha, Peter Daszak, Kevin J. Olival, Supaporn Wacharapluesadee

**Affiliations:** 1 EcoHealth Alliance, New York, NY USA; 2 Department of Biology, Faculty of Science, Ramkhamhaeng University, Bangkok, Thailand; 3 Wildlife Conservation Society, Viet Nam Country Program, Ha Noi, Viet Nam; 4 Wildlife Conservation Society, Health Program, Bronx, NY USA; 5 Division of Infectious Diseases, Faculty of Medicine, Thai Red Cross Emerging Infectious Diseases Clinical Centre, King Chulalongkorn Memorial Hospital, Chulalongkorn University, Bangkok, Thailand; 6 Loei Hospital, Loei, Thailand; 7 The Office of Disease Prevention and Control 5, Ratchaburi, Thailand; 8 Wat-Luang Health Promoting Hospital, Phanat Nikhom, Chonburi, Thailand; 9 Thai Red Cross Emerging Infectious Diseases-Health Science Centre, Faculty of Medicine, World Health Organization Collaborating Centre for Research and Training On Viral Zoonoses, Chulalongkorn Hospital, Chulalongkorn University, Bangkok, Thailand; 10 The Henry M. Jackson Foundation for the Advancement of Military Medicine, Bethesda, MD USA; 11 Thai Red Cross Emerging Infectious Diseases Clinical Centre, King Chulalongkorn Memorial Hospital, Bangkok, Thailand

**Keywords:** Surveillance, Behavioral surveillance, Zoonotic risk, Human–animal interaction, Risk perception, Coronavirus, Paramyxovirus, Flavivirus, Influenza, Enterovirus

## Abstract

**Background:**

Interactions between humans and animals are the key elements of zoonotic spillover leading to zoonotic disease emergence. Research to understand the high-risk behaviors associated with disease transmission at the human-animal interface is limited, and few consider regional and local contexts.

**Objective:**

This study employed an integrated behavioral–biological surveillance approach for the early detection of novel and known zoonotic viruses in potentially high-risk populations, in an effort to identify risk factors for spillover and to determine potential foci for risk-mitigation measures.

**Method:**

Participants were enrolled at two community-based sites (n = 472) in eastern and western Thailand and two hospital (clinical) sites (n = 206) in northeastern and central Thailand. A behavioral questionnaire was administered to understand participants’ demographics, living conditions, health history, and animal-contact behaviors and attitudes. Biological specimens were tested for coronaviruses, filoviruses, flaviviruses, influenza viruses, and paramyxoviruses using pan (consensus) RNA Virus assays.

**Results:**

Overall 61/678 (9%) of participants tested positive for the viral families screened which included influenza viruses (75%), paramyxoviruses (15%), human coronaviruses (3%), flaviviruses (3%), and enteroviruses (3%). The most salient predictors of reporting unusual symptoms (i.e., any illness or sickness that is not known or recognized in the community or diagnosed by medical providers) in the past year were having other household members who had unusual symptoms and being scratched or bitten by animals in the same year. Many participants reported raising and handling poultry (10.3% and 24.2%), swine (2%, 14.6%), and cattle (4.9%, 7.8%) and several participants also reported eating raw or undercooked meat of these animals (2.2%, 5.5%, 10.3% respectively). Twenty four participants (3.5%) reported handling bats or having bats in the house roof. Gender, age, and livelihood activities were shown to be significantly associated with participants’ interactions with animals. Participants’ knowledge of risks influenced their health-seeking behavior.

**Conclusion:**

The results suggest that there is a high level of interaction between humans, livestock, and wild animals in communities at sites we investigated in Thailand. This study highlights important differences among demographic and occupational risk factors as they relate to animal contact and zoonotic disease risk, which can be used by policymakers and local public health programs to build more effective surveillance strategies and behavior-focused interventions.

**Supplementary Information:**

The online version contains supplementary material available at 10.1186/s12879-022-07439-7.

## Introduction

Thailand is located in one of the major hotspots for emerging or re-emerging zoonotic diseases in Southeast Asia [1]. The high biodiversity and complex dynamic social and ecological environment in Thailand have placed a heavy burden of zoonotic and vector-borne diseases on the local population [2]. Chikungunya virus [3], Dengue virus [4], Japanese encephalitis virus [5], and rabies virus [6] continue to re-emerge and be endemic in Thailand. Human cases of highly pathogenic avian influenza (HPAI) A H5N1 were reported in 2004 in Thailand associated with poultry contact [7], and Nipah virus that has caused human disease outbreaks in South and Southeast Asia has been identified in local bat populations although with no known livestock or human cases [8, 9]. Even though overall burden of infectious diseases has been decreasing in recent decades [10], Thailand faces a significant threat from imported emerging zoonotic coronaviruses such as the severe acute respiratory syndrome coronavirus (SARS-CoV), the Middle East respiratory syndrome coronavirus (MERS-CoV), and the severe acute respiratory syndrome coronavirus 2 (SARS-CoV-2) given its role as a hub for travel and trade, including medical tourism [11]. Meanwhile, a large number of novel viruses from viral families known to harbor zoonoses are being discovered from wild animals in Thailand, representing a potential risk for emergence in humans [12].

Risk factors associated with zoonotic disease emergence in human populations typically result from the interactions among humans, animal hosts, and the environment, and are driven by dynamic changes in the social and ecological environments [13, 14]. In Thailand, ecological risk factors include forest conversion for agricultural cultivation [15, 16], intensive animal production [17], wildlife trade [18] and increasing human density and human movement [19–21] that create interfaces between humans and animals and affect pathogen transmission patterns. Among these, human contact with animals, directly or indirectly, is considered as the proximate risk factor for zoonotic pathogen transmission leading to emerging infectious diseases [22, 23]. However, despite the likely high rates of contact between local communities and animals that harbor potential zoonotic pathogens in Thailand [24–27], research to understand these high-risk behaviors associated with pathogen transmission and human–animal contacts is limited.

Here we applied an integrated behavioral–biological surveillance strategy to identify biological evidence of zoonotic spillover of viruses of epidemic or pandemic potential among the at-risk population, and to analyze the behavioral and other factors associated with the risk of zoonotic disease emergence. Our ‘One Health’ surveillance approach was designed to inform targeted risk-mitigation measures on identified risk factors based on the local context.

## Methodology

### Research location and target population

All biological surveillance and behavioral research was conducted under the USAID PREDICT-2 project from 2017 to 2018. Participants were recruited from both community and hospital sites to identify evidence of, and risk factors for, zoonotic disease exposure using community-based surveillance and clinic/hospital-based syndromic surveillance approaches. For community-surveillance, participants included those who are highly exposed to wildlife specifically bats, rodents or non-human primates in community settings through hunting, butchering or general handling within the context of their living or working environments. For clinic-based syndromic surveillance, pariticipants were patients who came to clinics presenting with disease symptoms of severe/acute respiratory illness (SARI/ARI); Influenza-like illness (ILI); fever of unknown origin (FUO); encephalitis; hemorrhagic fever; or diarrhea in combination with any of the previously mentioned illnesses of unknown etiology.

The community sites were chosen in areas with the presence of large bat colonies, contact with human populations via guano collection and tourism, and that had extensive poultry and swine production. With these criteria, two community sites in peri-urban areas were selected in Chonburi province (Eastern) and Ratchaburi province (Western) Thailand where potential zoonotic viruses have also been identified in the bat and rodent populations [8, 9, 28–31]. Local residents over the age of 12 who live, work, or visit (for examples—visit family, for religious reasons, holiday/vacation, go to hospital, go to market) these two community sites were eligible for enrollment in this study. About 91% of our study participants lived or work near the sites.

**Fig. 1:**
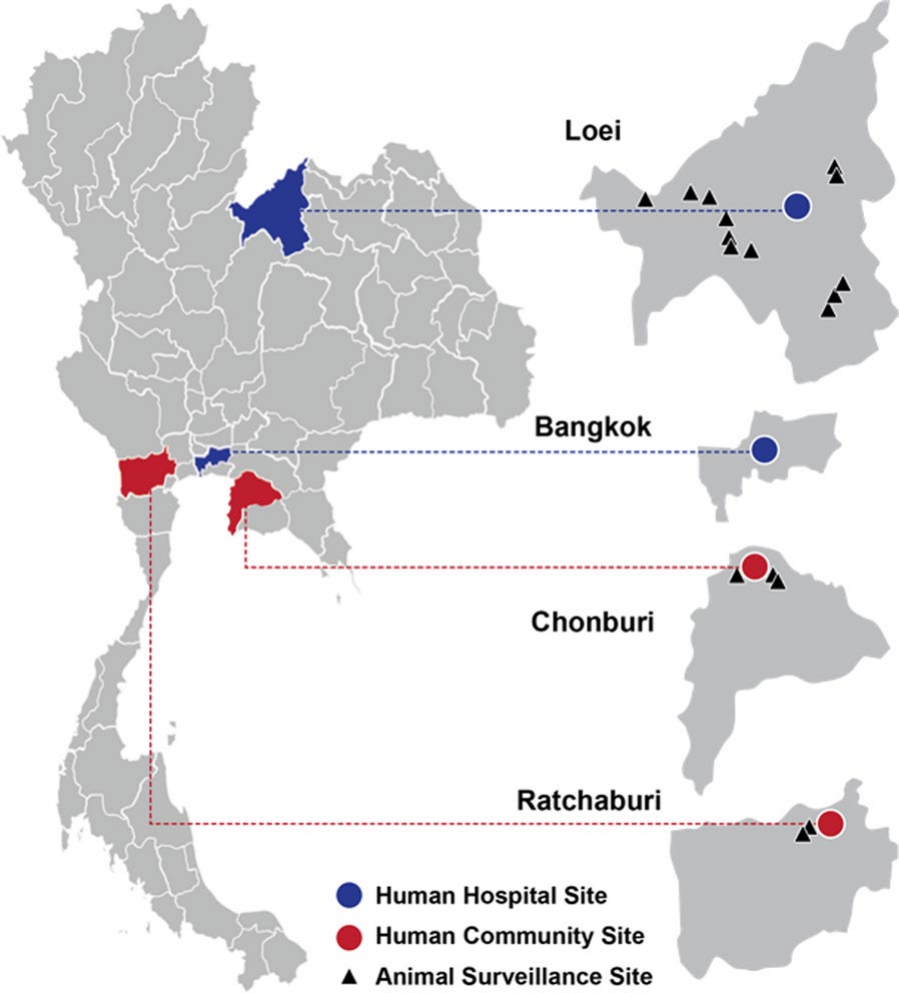
Two community sites and two hospital sites for human biological–behavioral surveillance in Thailand.Animal surveillance was conducted concurrently at three sites. (Authors’ own figure)

One clinical site was identified in Loei province in northeastern Thailand, which is located in the same province where viral surveillance among rodents and bats was also being conducted by our team (part of a separate study). A second clinical site was identified in Bangkok, central Thailand to bring in participants fitting our clinical descriptions from a wide geographic catchment area—sites did not necessarily overlap with community-based or animal sampling sites (Fig. 1).

### Recruitment and informed consent

Introductory visits were made by the study staff to each of the selected sites prior to the commencement of the study. Community visits began with discussions with local authorities and community leaders to introduce the study, followed by community meetings to discuss study details with community members, including: the voluntary nature of participation in the study, inclusion and exclusion criteria, as well as future dates, times, and locations relevant to study participation. At hospital sites, a study description letter was shared among relevant healthcare staff after meetings with hospital administration staff. Patients who were eligible for enrollment were identified by collaborating hospital staff during the standard intake procedures, from the overnight intake logs, the emergency room/ward, or the intensive care unit of the hospital, according to the study inclusion/exclusion criteria. Participants who met the criteria for enrollment at hospital sites, together with their parents or legal guardians if applicable, were invited to speak with the study staff regarding the details of the study, to review the study information and informed consent form, and ask any relevant questions. All study documents were in Thai and study team members were fluent in local languages, ensuring the participants fully understood the study and procedures.

In community sites, children less than 12 years were not enrolled in the study due to the fact that they were likely not highly exposed to wildlife through hunting, butchering or general handling. Children aged 12 to 17 years were eligible to participate in the study if they provided assent and were accompanied by a parent or legal guardian who provided informed consent and remained present during the entire consent process. In hospital sites, children younger than 12 years were eligible to participate at hospital sites with the informed consent of a parent or legal guardian. Only consented participants were enrolled in the study. Participation in the study was completely voluntary, and all participants were informed that they could withdraw from the study at any time without consequences.

### Data collection and management

Biological and behavioral questionnaire data were collected from individuals who met recruitment and inclusion criteria and consented. Data collection was conducted annually at the two community sites in 2017 and 2018, respectively, while continuous data collection was performed at hospital sites from May 2017 to November 2018. A standardized questionnaire (Additional file 1), developed to be consistent across multiple countries and cultural contexts, was administered to understand the demographics, living conditions, health history, and animal contact behaviors and attitudes of the participants [32]. Biological specimens were collected from each participant who completed the questionnaire. At community sites, oral swabs or nasopharyngeal swabs, whole blood, and urine specimens were collected from each participant. At the hospital sites, nasopharyngeal swabs, rectal swabs, whole blood, and urine specimens were collected from each participant. Nasopharyngeal swabs and other swabs were collected by using flocked swabs (Copan Flock Technologies SRL, Italy) and rayon-tipped swabs, respectively (Puritan, USA). Swab specimens were collected in trizol and viral transport medium.

Specimens collected from community sites were temporarily kept on ice after collection and transported to be stored in ultralow freezers at − 80 °C within one day. The collected specimens at hospital sites were directly stored in the dedicated ultralow freezers at − 80 °C. Cold chains were maintained for the transportation of specimens to the project laboratory facility for testing.

### Lab methods and molecular screening

The nucleic acid was extracted from collected specimens using the high-throughput Biomerieux automated nucleic acid extraction (bioMérieux, France) per manufacturer instruction with positive and negative controls to validate the procedure. RNA was reverse-transcribed using SuperScript III First-strand cDNA synthesis kit (Invitrogen, USA). A detailed description of protocols for screening all samples using pan RNA virus assays is provided in the Laboratory Protocols for PREDICT II Surveillance Version 2: 2016-05 [33]. Specimens collected at the hospital and community sites were tested for coronaviruses [34, 35], filoviruses [36], flaviviruses[37], influenza viruses [38], and paramyxoviruses [39] by conventional PCR using Invitrogen Platinum TAQ DNA polymerase kit on a PCR Thermal Cycler machine. Additional tests were performed on the samples collected at hospital sites for alphaviruses [40], arenaviruses [41], orthobunyaviruses [42], rhabdoviruses (Unpublished Designed at CII, Laboratory Protocols for PREDICT Surveillance Version 2: 2013-03) hantaviruses [43], other-enteroviruses with conventional PCR [44] when the clinical history was relavant to the virus infection.

### Data analysis

We first conducted descriptive analysis to determine significant associations between demographic factors (gender, age, education, primary livelihood, length of time living at current location, number of people living with), living environment and practices (drinking water treated, food storage), travel history, animal contact and self-reported symptoms in the past year as independent variables, and PCR confirmed diagnoses as the outcome. For univariate descriptive analyses, we used Chi-square and Fisher’s exact tests performed using STATA/IC version 16.0 (StataCorp., College, TX, USA). The association between demographic factors, attitudes and health behavior around animal contacts as independent variables and animal contact as the outcome was also assessed. Further, the association of participants’ demographic factors with their perception of risk from animal contacts was assessed. A p-value < 0.05 was considered statistically significant. Categorical variables between groups were compared by Chi-square test or Fisher’s exact test and t-test.

To rank the relative importance of multiple predictor variables in our study, we fit a least absolute shrinkage and selection operator (LASSO) regression to characterize associations between: (1) demographics, living conditions, and animal contact as predictor variables, and self-reported unusual symptoms in the past year as the outcome and (2) demographics as the predictor variable and animal contact as the outcome.

The LASSO regression is an adaptation of the generalized linear model (GLM) and was selected because it is effective at minimizing prediction error for datasets with many predictor variables. The model identifies subsets of predictors that are associated with the outcome of interest by applying a shrinkage operation to regression coefficients and shrinking some coefficients to exactly zero [45]. Demographic variables, living environment, and practices were included in the model as independent and interaction terms in order to account for potential confounding. Because the LASSO does not generate confidence intervals, we repeated the model using bootstrapping to calculate bootstrap support, i.e., the proportion of times a predictor variable is selected in the model [45–48]. LASSO regressions were run using the glmnet package in R version 3.6.0 [49].

### Ethics statement

The study protocols were reviewed and approved by the Institutional Review Board of Chulalongkorn University (No. 380/59); and the Institutional Review Board Administration of the University of California, Davis (No. 804522-20).

## Results

### Characteristics of participants and households

A total of 678 participants were enrolled in the study from April 2017 to November 2018 at community sites (n = 472) and hospital sites (n = 206). The majority of participants who enrolled at the hospital sites were males, young adults, and children compared to community sites where the majority were female, middle-aged and senior adults. Most participants (84%) had secondary school education or less. Primary livelihoods of the participants include crop production, extraction of mineral, gas, oil or timber, zoo and sanctuary animal care worker and other non-animal business such as protected area worker or migrant laborers. We also identified several participants with high-levels of repeated occupational exposure to animals at or near the surveillance site, including poultry and swine farm workers (n = 2), animal sanctuary staff (n = 34), and bat guano collectors (n = 29). The majority of the participants had been living at their current residences for more than five years (88%) and almost all participants shared living space with others (99%). Water infastructure was good at most sites, with > 90% of participants reporting drinking water from pipe or tap water. While the majority of participants (68%) treated water for drinking by filtering, boiling, or solar disinfection, about one third of them reported no further water treatment. Participants indicated having dedicated locations for human waste including well-maintained toilets and having used containers with covers for food storage (Table 1).

**Table 1:** Demographic characteristics of participants and households

	Community sites (n = 473)	Hospital sites (n = 205)	Total (n = 678)
Gender			
Female	314 (66%)	75 (37%)	389 (57%)
Male	159 (34%)	130 (63%)	289 (43%)
Age group (in years)^a^			
< 12	0 (0%)	54 (26%)	54 (8%)
12–19	16 (3%)	17 (9%)	33 (5%)
20–39	70 (15%)	66 (32%)	136 (20%)
40–59	200 (42%)	40 (19%)	240 (35%)
60–79	173 (37%)	27 (13%)	200 (30%)
> 80	13 (3%)	2 (1%)	15 (2%)
Education			
None	114 (24%)	28 (14%)	58 (9%)
Primary school	316 (67%)	113 (55%)	344 (51%)
Secondary school	31 (7%)	35 (17%)	166 (24%)
College/university/professional	2 (3%)	29 (14%)	110 (16%)
Primary livelihood^b^			
Non-animal business	219 (46%)	53 (26%)	272 (40%)
Crop production	63 (13%)	49 (24%)	112 (17%)
Child/student	16 (3%)	71 (35%)	87 (13%)
Homemaker/unemployed	61 (13%)	22 (11%)	83 (12%)
Extraction of minerals, timber and bat guano	52 (11%)	0 (0%)	52 (8%)
Zoo/sanctuary worker	34 (7%)	1 (0%)	34 (5%)
Construction worker	16 (3%)	5 (2%)	21 (3%)
Community clinic worker and traditional healer	10 (2%)	1 (< 1%)	11 (2%)
Government official	0 (0%)	2 (< 1%)	2 (< 1%)
Animal production business	1 (< 1%)	1 (< 1%)	2 (< 1%)
Non-timber forest product collector	1 (< 1%)	2 (< 1%)	2 (< 1%)
Length of time living at the location			
< 1 year	6 (1%)	11 (5%)	17 (3%)
1–5 years	12 (3%)	47 (23%)	59 (9%)
5–10 years	57 (12%	109 (53%)	166 (24%)
> 10 years	398 (84%)	38 (19%)	436 (64%)
No. of people living in the same dwelling			
None	2 (< 1%)	0 (0%)	2 (< 1%)
1–5	359 (76%)	144 (70%)	503 (74%)
5–9	106 (22%)	57 (28%)	163 (24%)
10 and above	6 (1%)	4 (2%)	10 (1%)
Sources of drinking water^b^			
Piped in water/water tap	448 (95%)	181 (88%)	629 (93%)
Covered well	21 (4%)	110 (54%)	31 (5%)
Uncovered well/pond/river	28 (6%)	8 (4%)	36 (5%)
Water truck/rainwater harvest	58 (12%)	30 (15%)	88 (13%)
Water is treated			
Yes	279 (59%)	181 (88%)	460 (68%)
No	194 (41%)	24 (12%)	218 (32%)
Dedicated location for human waste			
Yes	468 (99%)	196 (96%)	664 (98%)
No	5 (1%)	9 (4%)	14 (2%)
Containers for food storage in household^b^			
Yes, with covers	468 (99%)	197 (96%)	665 (98%)
Yes, without covers	1 (< 1%)	4 (2%)	5 (1%)
No	4 (1%)	4 (2%)	8 (1%)

^a^Children under age 12 were not eligible for enrollment at community sites

^b^Select all that apply to the question. Values are the percentages of total participants

### Molecular virus testing and associated risk factors

#### Laboratory diagnosis of viral infection

Specimens from 61 participants (9%) tested positive for known viruses from five viral families, including orthomyxoviruses (Influenza A and B viruses) (n = 46) [50], paramyxoviruses (Measles and Human Parainfluenza viruses) (n = 9), coronaviruses (OC43 and HKU1 coronaviruses) (n = 2), flaviviruses (Dengue and Zika viruses) (n = 2), and enteroviruses (Human Enteriovirus B) (n = 2). No novel viruses were discovered in any samples screened from participants. Molecular positivity for influenza viruses, paramyxoviruses, coronaviruses, flaviviruses and enteroviruses via RT-PCR in samples collected from our study population were 77%, 14.8%, 3.3%, 3.3%, and 3.3%, respectively. Sixty (98%) of the positive samples were collected from hospital participants. One (2%) sample collected from a participant at a community site was also found to be positive (human coronavirus HKU1) [51]. Co-infection of human parainfluenza virus and influenza A, partial subtype H1 was identified in one specimen (Table 2).

**Table 2:** Laboratory diagnosis of viral infection

Laboratory diagnosis of viral infections	No. of participants (n = 61)
Coronaviruses	2 (3.3%)
Beta-coronavirus 1 (OC43)	1 (2%)
Human coronavirus HKU1	1 (2%)
Influenza viruses	47 (77%)
Influenza A	3 (5%)
Influenza A, subtype H1N1	21 (34%)
Influenza A, subtype H3N2	11 (18%)
Influenza A, partial subtype H1	1 (2%)^a^
Influenza A, partial subtype N1	1 (2%)
Influenza B	10 (16%)
Paramyxoviruses	9 (14.8%)
Measles virus	1 (2%)
Human parainfluenza virus 1	8 (13%)^a^
Flaviviruses	2 (3.3%)
Zika virus	1 (2%)
Dengue virus serotype 2	1 (2%)
Enteroviruses	2 (3.3%)
Human enterovirus B	2 (3%)

^a^Co-infection of Human parainfluenza virus and Influenza A, partial H1 in one specimen

#### Characteristics and risk factors of laboratory diagnosis for known human pathogens

The majority of participants who had PCR-confirmed viral infections were male (67%). Participants younger than 10 years of age made up 43% of those who tested positive. The majority of PCR-positive participants were children or students (59%) and 18% were participants who worked in crop production. Most participants who tested positive reported living in their dwelling for 5–10 years (43%) whereas the majority of those who tested negative reported living in their dwelling for more than 10 years (69%). Those with a positive test were more likely to have travelled in the past year (67%) for different reasons such as to work, visit family, move to a new place, go to hospital/seek medical care, go to market or for religious reasons or holiday/vacation than those with a negative test (49%). There was no significant difference between participants with a positive test and those with a negative test in terms of the number of people present in their dwelling and whether they used a cover for food storage. A significantly higher number of participants with a positive test treated their drinking water compared to those with a negative test (95% vs 65%), but it might have been masked by the fact that higher proportion of hospital site participants treated their drinking water compared to community site participants regardless of the laboratory results. There was no significant difference in having any animal contact between participants with PCR-confirmed infections and those without. However, when we excluded dogs and cats, a significantly increased proportion of those with a positive test (72%) reported animal contact as compared to those with a negative test (57%) (Table 3).

**Table 3:** Characteristics and risk factors of PCR-confirmed diagnosis

	Positive test (n = 61)	Negative test (n = 617)	p-value
Gender			
Male	41 (67%)	248 (40%)	< 0.0001
Female	20(33%)	369 (60%)	
Age group in years			
< 10	26 (43%)	27 (4%)	< 0.0001
10–19	9 (15%)	25 (4%)	
20–39	19 (31%)	117 (19%)	
40–59	3 (5%)	237 (39%)	
60–79	4 (6%)	196 (32%)	
> 80	0 (0%)	12 (2%)	
Education			
None	20 (33%)	38 (6%)	< 0.0001
Primary school	19 (31%)	325 (53%)	
Secondary school	13 (21%)	153 (25%)	
College/university/professional	9 (15%)%)	101 (16%)	
Primary livelihood			
Child/student	36 (59%)	51 (8%)	< 0.0001
Construction	1 (2%)	20 (3%)	
Crop production	11 (18%)	101 (16%)	
Extraction of minerals, gas, oil, timer	1 (2%)	51 (8%)	
Homemaker/unemployed	2 (3%)	81 (13%)	
Non-animal business	9 (15%)	263 (43%)	
Military	1 (2%)	1 (< 1%)	
Zoo/sanctuary animal health care	0 (0%)	34 (5%)	
Rancher/farmer animal production business	0 (0%)	2 (< 1%)	
Nurse, doctor, traditional healer, community health worker	0 (0%)	11 (2%)	
Forager/gatherer/non-timber forest product collector	0 (0%)	2 (< 1%)	
Length of time living at location (in years)			
< 1	5 (8%)	12 (2%)	< 0.0001
1–5	19 (31%)	40 (6%)	
5–10	26 (43%)	140 (23%)	
> 10	11 (18%)	425 (69%)	
Travelled			
Yes	41 (67%)	304 (49%)	0.007
No	20 (33%)	313 (51%)	
Food storage for the household			
Yes, with covers	58 (95%)	607(98%)	0.06
Yes, without covers	2 (3%)	3 (< 1%)	
No	1 (2%)	7 (1%)	
Drinking water treated			
Yes	58 (95%)	402 (65%)	< 0.0001
No	3 (5%)	215 (35%)	
Number of people in dwelling			
None	0 (0%)	2 (< 1%)	0.07
1–5	38 (62%)	465 (75%)	
6–9	21 (34%)	142 (23%)	
10 and above	2 (3%)	8 (1%)	
Having animal contact			
Yes	54 (89)	561 (91%)	0.54
No	7 (11%)	56 (9%)	
Having animal contact excluding dogs and cats			
Yes	44 (72%)	352 (57%)	0.02
No	17 (28%)	265 (43%)	
Self-reported unusual symptoms last year			
Yes	29 (48%)	189 (31%)	0.007
No	32 (52%)	428 (69%)	
Symptoms last year in other people you live with			
Yes	20 (33%)	103 (17%)	0.002
No	41 (67%	514 (83%)	

#### Self-report unusual symptoms in the past year

Participants were asked if they had symptoms in the past year that they considered unusual, i.e., any illness or sickness that is not known or recognized in the community, including by medical or treatment providers. A significantly higher proportion of participants who tested positive reported having unusual symptoms (48%) or observed symptoms in people they lived with (33%) compared to those with negative PCR tests in the year prior to this study (Table 3).

**Fig. 2:**
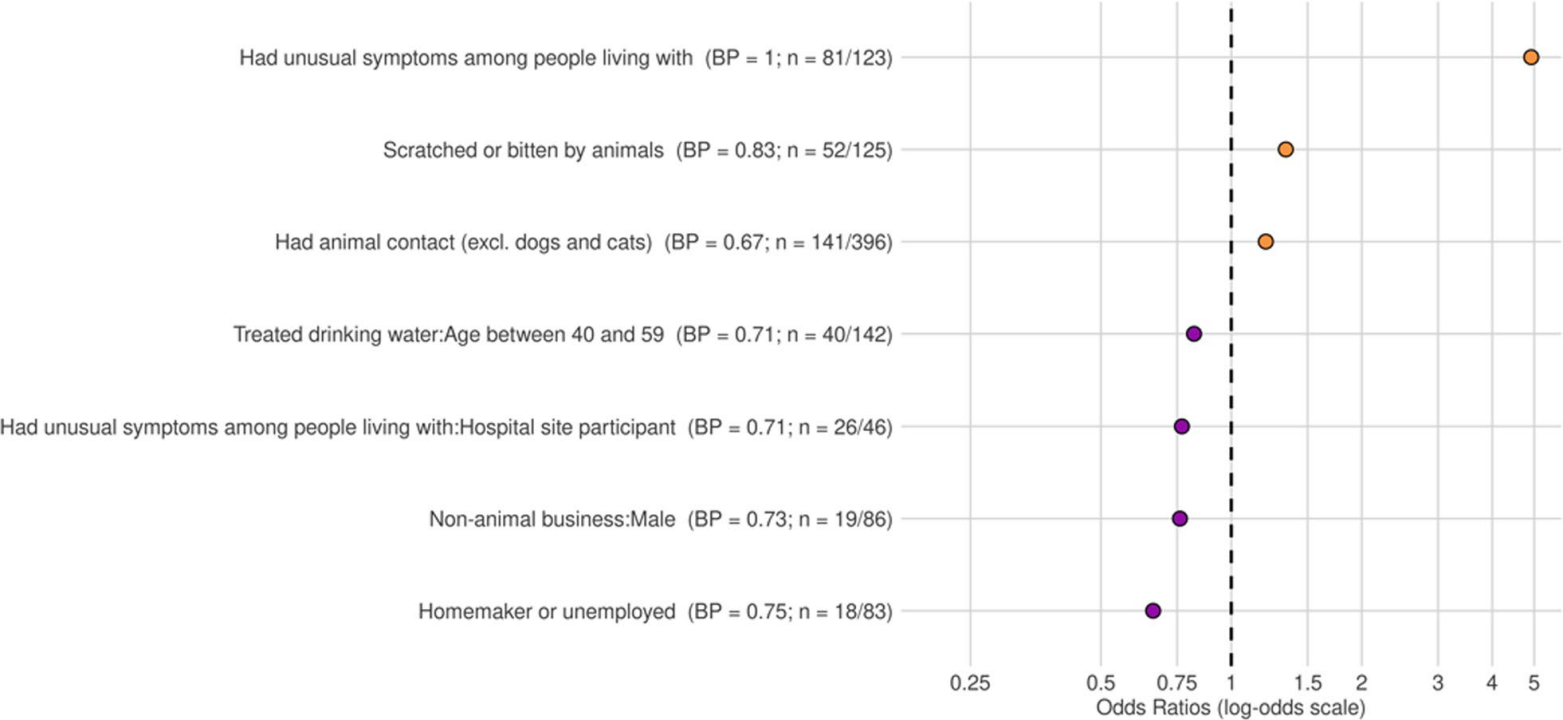
Most salient predictors of self-report unusual symptoms in the past year. *BP*  bootstrap support, *n*  count positive. Bootstrap support values $\ge$ 0.6 are reported here, meaning they were identified as associated with the outcome for 60% or more of the bootstrap iterations. Odds ratio > 1 are positively associated with the outcome, and odds ratio < 1 are negatively associated with the outcome

The LASSO regression identified three factors with a positive association for reporting unusual symptoms in the past year: having other household members who had unusual symptoms (OR = 4.92; bootstrap support (BP) = 1), being scratched or bitten by animals in the same year (OR = 1.34; BP = 0.83), and having animal contacts excluding dogs and cats (OR = 1.20; BP = 0.67). Additional demographic factors associated with being less likely to report unusual symptoms in the past year were: participants aged between 40 and 59 who had their drinking water treated (OR = 0.82; BP = 0.71), male participants who worked in the non-animal business for their livelihoods (OR = 0.76; BP = 0.73), and those who were unemployed or identified themselves as a homemaker (OR = 0.66; BP = 0.75). Interestingly, those whose household members had symptoms in the previous year and were specifically recruited at the hospital site were less likely to report unusual symptoms themselves in the same year (OR = 0.77; BP = 0.71) (Fig. 2).

### Human–animal interaction among participants

#### Animal contact in life time

Almost all participants reported having contacts with animals in their lifetime (91%). Most participants have had household pets (82%), raised animals (70%), had animals other than pets come into their houses (66%) or handled animals (65%). In addition, participants reported having been scratched or bitten by animals (34%), cooked meat (33%), eaten raw meat (19%), slaughtered animals (19%), hunted animals (17%), eaten food after animals touched it (11%), shared water with animals (9%), and had animal feces near their food (8%). Seven (1%) participants reported having eaten sick animals, seven (1%) reported having found or collected dead animals, and five (< 1%) reported having sold dead animals in their lifetime (Additional file 2: Fig. S1).

With the exclusion of dogs and cats, 58% of participants reported contacts with animals and the animals with whom they most commonly had contact were poultry (31%), followed by rodents (20%), swine (19%), cattle (16%), and birds (8%). 24 participants reported contacts with bats (4%) and 11 had contacts with carnivores (2%). Out of the 29 bat guano miners identified in the study, only six of them reported contacts with bats (1%). No participants reported having contacts with pangolins or camels (Additional file 2: Fig. S2).

#### Specific forms of animal contact in the past year

**Fig. 3:**
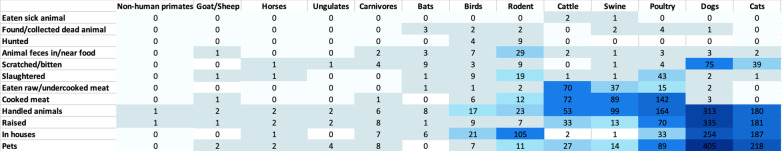
Animal contact activities in the past year. *Numbers in cells indicate the number of participants

To further understand details around animal contacts, participants were asked about the types of animals they came into contact within the past year and the nature of the interaction. Many participants reported raising and handling poultry, swine, and cattle and a few reported eating the raw or undercooked meat of these animals. Seeing rodents in the house was common among the study participants and participants reported seeing rodents’ feces in or near their food. Additionally, some participants reported handling bats, having bats in the house, or being scratched or bitten by them (Fig. 3).

#### Demographics, attitudes, and behaviors around human–animal contact

Of the study population who had any animal contact in their lifetime, 57% were female and 43% were male. Age and education level of the participants were significantly associated with having animal contact. Participants aged 40–59 years most frequently reported having animal contact followed by the 60–79 and 20–39 year-old age groups. A higher percentage of participants with primary school education or less reported having animal contacts compared to those with college/university/professional education. Participants’ livelihood, concern about disease outbreaks in live animal markets, knowledge about the risk of open wounds, or choice of action when bitten or scratched by animals were not associated with whether they had animal contact (Table 4).

**Table 4:** Demographics, attitudes and behaviors around human–animal contact

	Animal contact (n = 615)	No animal contact (n = 63)	p-value	Animal contact (exclu dogs and cats) (n = 396)	No animal contact (exclu dogs and cats) (n = 282)	p-value
Gender						
Male	265 (43%)	24 (38%)	0.45	194 (49%)	95 (34%)	< 0.0001
Female	350 (57%)	39 (62%)		202 (51%)	187 (66%)	
Age group (in years)						
< 10	45 (7%)	8 (13%)	< 0.0001	34 (7%)	19 (13%)	0.003
10–19	32 (5%)	2 (3%)		14 (5%)	20 (3%)	
20–39	132 (21%)	4 (6%)		90 (21%)	46 (6%)	
40–59	225 (37%)	15 (24%)		149 (37%)	91 (24%)	
60–79	170 (28%)	30 (48%)		105 (28%)	95 (48%)	
> 80	8 (2%)	4 (6%)		3 (2%)	9 (6%)	
Education						
None	52 (8%)	6 (10%)	0.048	34 (9%)	24 (8%)	0.78
Primary school	303 (49%)	41 (65%)		199 (50%)	145 (51%)	
Secondary school	154 (25%)	12 (19%)		94 (24%)	72 (25%)	
College/university/professional	106 (17%)	4 (6%)		69 (17%)	41 (15%)	
Primary livelihood						
Child/student	79 (13%)	8 (13%)	0.58	53 (13%)	34 (12%)	< 0.0001
Construction	21 (3%)	0 (0%)		8 (2%)	13 (5%)	
Crop production	105 (17%)	7 (11%)		83 (21%)	29 (10%)	
Extraction of minerals, gas, oil, timber	48 (8%)	4 (6%)		29 (7%)	23 (8%)	
Homemaker/unemployed	75 (12%)	8 (13%)		58(15%)	25 (9%)	
Non-animal business	239 (39%)	33 (52%)		128 (32%)	144 (51%)	
Military	2 (< 1%)	0 (0%)		2 (< 1%)	0 (0%	
Zoo/sanctuary animal health care	32 (5%)	2 (3%)		25 (6%)	9 (3%)	
Rancher/farmer animal production business	2 (< 1%)	0 (0%)		2 (< 1%)	0 (0%)	
Nurse, doctor, traditional healer, community health worker	10 (2%)	1 (1%)		6 (1%)	5 (2%)	
Forager/gatherer/non-timber forest product collector	2 (< 1%)	0 (0%)		2 (< 1%)	0 (0%)	
Worried about diseases/outbreaks in live animals in local market						
Yes	332 (54%)	27 (43%)	0.09	212 (54%)	147 (52%)	0.72
No	283 (46%)	36 (57%)		184 (46%)	135 (48%)	
Risk of open wounds						
Yes	201 (32%)	14 (22%)	0.13	114 (29%)	101 (36%)	0.06
No	299 (49%)	32 (51%)		208 (19%)	58 (20%)	
Don’t know	115 (19%)	17 (27%)		74 (52%)	123 (44%)	
Action taken when scratched or bitten^a^						
Visit doctor	218 (51%)	20 (61%)	0.89	121(45%)	117 (62%)	0.001
Wash wound with soap and water	91 (21%)	5 (15%)		65 (24%)	31 (16%)	
Rinse wound with water	24 (6%)	2 (6%)		21 (8%)	5 (3%)	
Bandage wound	42 (10%)	3 (9%)		24 (9%)	21 (11%)	
Nothing—kept working	50 (12%)	3 (9%)		38 (14%)	15 (8%)	

^a^Excluded participants who had never been scratched or bitten

Gender, age and occupation were associated with having contact with animals that were not dogs and cats. Higher proportion of males reported having animal contact compared to that of females and those in the 40–59-year age group most reported animal contact. Fewer participants with animal contact answered that they would visit a doctor when scratched or bitten by animals (45%) compared to those with no animal contacts (62%). More participants with animal contact said they would continue working after an animal bite or scratch (14%), compared to those with no animal contact (8%) (Table 4).

**Fig. 4:**
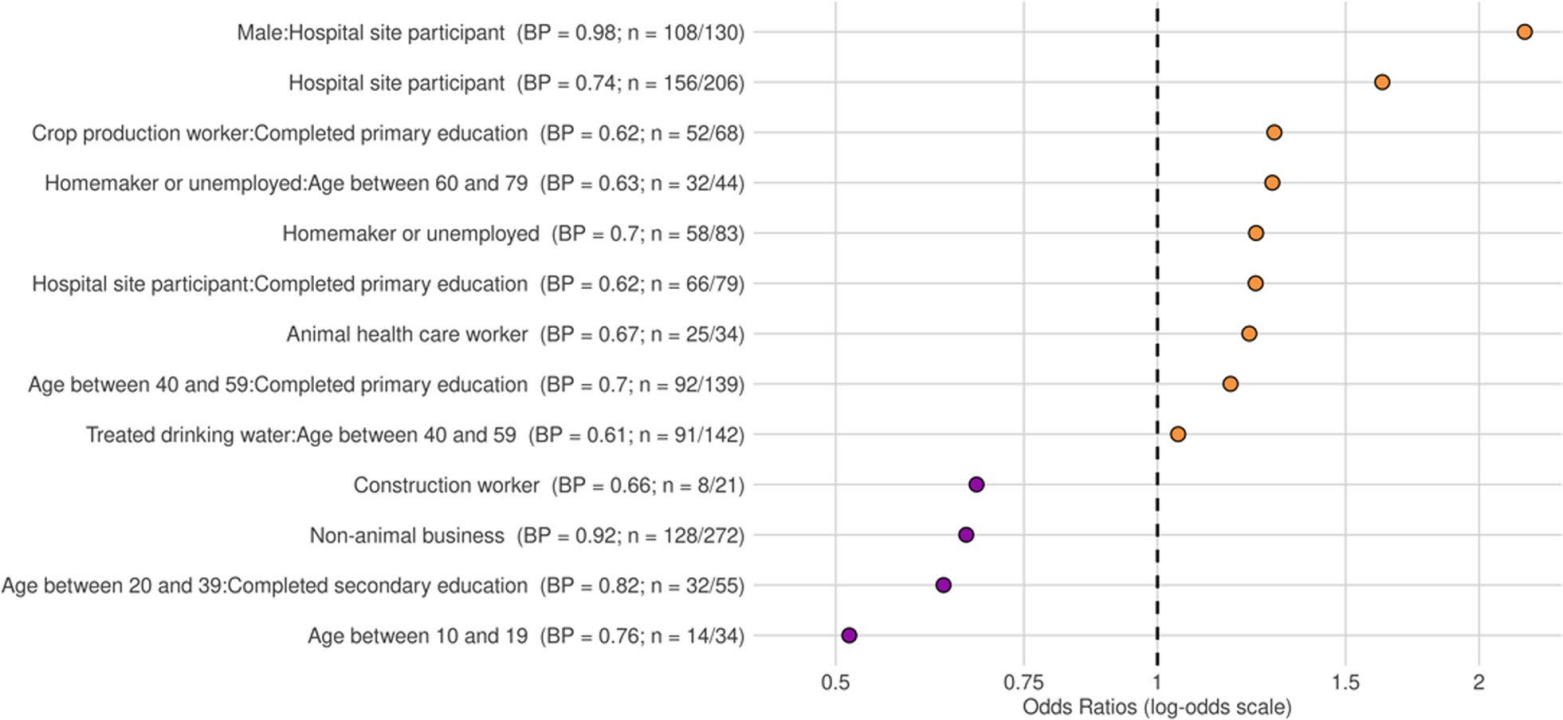
Most salient predictors of having animal contact excluding dogs and cats. *BP* bootstrap support, *n* count positive. Bootstrap support values $\ge$ 0.6 are reported here, meaning they were identified as associated with the outcome for 60% or more of the bootstrap iterations. Odds ratio > 1 are positively associated with the outcome, and odds ratio < 1 are negatively associated with the outcome

The LASSO regression showed that participants recruited at hospital sites, particularly males (OR = 2.21; s = 0.98) or those who completed no more than their primary school education (OR = 1.24; s = 0.62) were more likely to have contact with animals other than dogs and cats. Other groups of participants who were more likely to report animal contacts were crop production workers who had not gone beyond primary school (OR = 1.29; 0.62), those aged between 40 and 59 who had not gone beyond primary school (OR = 1.17; BP = 0.7), crop production workers who had not gone beyond primary school (OR = 1.29; BP = 0.62), animal health care worker (OR = 1.22; BP = 0.67) and homemakers or unemployed participants who were aged 60–79 (OR = 1.28; BP = 0.63). Those with livelihood activities such as working in construction (OR = 0.68; BP = 0.66) or non-animal related businesses (OR = 0.66; BP = 0.92) were less likely to report having animal contact. Moreover, those aged between 10 and 19 (OR = 0.51; BP = 0.76) and those aged between 20 and 39 who completed their secondary education (OR = 0.63; BP = 0.82) were less likely to report animal contact (Fig. 4).

### Perception of risks from live animal market

More female participants (63%) were concerned about disease outbreaks in live animal markets compared to male participants (37%). There was not a significant difference in age groups among participants who said they were worried about disease outbreaks compared to participants who did not. Those who said they were worried about disease outbreaks also said there are risks of having open wounds when slaughtering or butchering (44%) but those who were not worried about outbreaks reported that there was no risk of open wounds while slaughtering or butchering (61%). Regarding actions taken when scratched or bitten, the majority of both groups said they would visit a doctor (Table 5). Similarly, significantly higher numbers of those who lacked knowledge of the risk of having open wounds and those who said there was no risk even with open wounds also said that they would keep working with animal’s scratches or bites compared to those who had knowledge of risk of open wounds (12% and 17% respectively vs 6%) (data not shown).

**Table 5:** Perception of risks from live animal market

	Worried about diseases/outbreak in live animals in local market		
	Yes (N = 359)	No (N = 319)	p-value
Gender			
Male	132 (37%)	157 (49%)	0.001
Female	227 (63%)	162 (51%)	
Education			
None	39 (11%)	19 (6%)	0.05
Primary school	184 (51%)	160 (50%)	
Secondary school	77 (22%)	89 (28%)	
College/university/professional	59 (16%)	51 (16%)	
Age group (in years)			
< 20	48 (14%)	39 (12%)	0.46
20–39	73 (20%)	63 (20%)	
40–59	133 (37%)	107 (33%)	
60–79	99 (28%)	101 (32%)	
> 80	4 (1%)	8 (3%)	
Risk of open wounds			
Yes	157 (44%)	58 (18%)	< 0.0001
No	138 (38%	193 (61%)	
Don’t know	64 (18%)	68 (21%)	
Action taken when scratched or bitten^a^			
Visit doctor	135 (54%)	103 (49%)	0.85
Wash wound with soap and water	49 (20%)	47 (22%)	
Rinse wound with water	13 (5%)	13 (6%)	
Bandage wound	23 (9%)	22 (10%)	
Nothing—kept working	28 (11%)	25 (12%)	

^a^Excluded participants who had never been scratched or bitten

## Discussion

Our study provides important insight into community-level animal contact and detailed descriptions of livelihood activities and zoonotic disease risk perception from Thailand in a period (2017–18) just prior to major behavioral changes during COVID-19. Our findings contribute to much-needed human behavioral data and social science approaches to identify pathways of transmission of viruses from animals to humans in a non-outbreak condition and suggest pathways for targeted interventions and prevention strategies. We integrated a quantitative behavioral survey into virus surveillance among populations living and working at high-risk human–animal interfaces in Thailand, to identify the behavioral drivers that are associated with zoonotic disease emergence and transmission. Vector-borne diseases and viral pathogens of zoonotic potential were detected in the study, including several human-to-human transmitted pathogens detected via clinical surveillance. We identified associated risk factors in self-report symptoms, demographics, and behaviors, attitudes and risk perceptions around animal contact. These findings provide insights into the risk factors for zoonotic spillover and guidance for targeted zoonotic disease surveillance and behavioral change strategies for zoonotic risk mitigation in Thailand.

### Viral detection and undiagnosed symptoms

Viruses detected by molecular assays included human CoVs, Influenza A, Influenza B, Human Parainfluenza Virus, Measles virus, Zika virus, Dengue-2 virus, and Human Enterovirus-B from hospital sites in Northeastern and central Thailand and community sites in eastern and western Thailand. Some of these pathogens are zoonotic and shared between people and animals such as cattle, birds, and swine [52]. Zika and dengue virus infections detected in this study are reported throughout the year in Thailand provinces. The prevalence of human coronaviruses we found was very similar to numbers cited in multiple studies of the same region [53–55]. Studies cite Influenza A of any type, as the most common cases among all Asia–Pacific countries, which is reflected in our study results [56, 57]. We also found that of Influenza A subtypes, A(H1N1) was the most prevalent followed by A(H3N2); results that agreed with other studies looking at Influenza activity in Thailand [56–58]. We found 9 participants to be positive for Paramxyoviruses (1 Measles, 8 Human Parainfluenza virus 1), but no evidence of any novel or zoonotic paramyxovirus infection. Our surveillance included participants living or working in close proximity to a large fruit bat colony (Pteropus lylei) where Nipah virus has been detected [59], yet we did not find any positive human samples among our study participants. Additional targeted surveillance including larger enrollment sizes, serological surveys, and/or longitudinal surveillance of people living in close association with wildlife populations may yield additional insights about zoonotic virus exposure and prevelance at these sites.

Although all 205 participants enrolled at the hospital sites had been clinically diagnosed with symptoms such as fever, headache, chills, joint pain, muscle pain, convulsions, diarrhea, vomiting, malaise or altered consciousness, only 60 participants (30%) had confirmed positive laboratory results in this study. Since routine diagnoses were conducted at the hospital sites to rule out known bacterial or other common infections, it is possible that these undiagnosed participants may have contracted other untested or unknown pathogens, including zoonotic pathogens which are often reported in people who work with or have frequent contact with animals [22, 23]. Further investigations to identify the causative agents of these clinical cases should include unbiased methods for viral amplification and metagenomic sequencing [60].

Despite the fact that most of the virus detected in this study are human to human transmitted viruses, we cannot rule out zoonotic infections as a cause of illness in the 70% of clinical cases with no detected pathogen in our target viral families. First, over 90% of our study population had animal contact in their lifetime with hospital sites participants reporting to have more animal contact than those from community sites. In addition, 24% of our study population work in the crop production business in which they might have frequent contact with rodents or other wildlife, and livestock in the crop fields and were exposed to manure from poultry, cattle, bats, and swine in the form of fertilizers. Another possible reason for laboratory unconfirmed infections is that the pathogen responsible for illness may have been cleared in the time before testing, hence rendering molecular testing ineffective. Moreover only one viral infection (from a known human pathogen, human coronavirus HKU1) was found in the community participants who had high levels of exposure to wildlife. Several factors could explain this, including that participants did not have any current infections at the time of the study or any asymptotic infections the community participants might have been by viral pathogens that were not covered by our molecular diagnostic assays. In these cases, it is important to pair molecular and serological assays for a broader diagnosis and to utilize new serological platforms for multiplexing to broadly identify exposure to multiple related pathogens [61]. In addition, all subtypes of influenza A and H3N2, some of which our study detected, had zoonotic potential. However, only with whole genome sequencing and comparative analyses with data obtained from poultry, swine, wild birds and others from the community will we be able to tell if these were zoonotic or human transmitted infections.

Many factors contribute to the emergence and spread of infectious diseases in different geographic areas and populations, including travel and human movement [62], which may involve short distances or crossing provinces or international borders. In our study, we found that those with viral infections had traveled more frequently than those with a negative test. Extensive domestic or international travel might result in a pathogen which could be transmitted directly or indirectly to another person or acquired infections from those places visited that could be brought back to participants households and/or local population when they returned.

### Exposure to zoonotic pathogen through human-animal interactions

While human-animal interactions are a primary risk factor for zoonotic disease emerge [1, 13, 14, 22, 23], the specific interactions and behaviors that lead to zoonoses exposure are less understood. In the absence of serology tests in our study, participants were asked to report symptoms in the past year to assess the association with different behaviors within the high-risk communities. While self-reported data have obvious biases, these approaches have been widely used in disease surveillance and risk factors studies [63–65]. Our study showed that having animal contacts, excluding dogs and cats, and having been scratched or bitten by animals in the past year were significantly associated with having unusual symptoms in the same year and potential viral infections. These findings are corroborated by other previously published studies [64–68]. Household transmission of infections were observed as our data showed that those whose household members reported unusual symptoms in the past year were more likely to report symptoms themselves. However, this finding seems to be specific to community site participants since participants recruited at the hospital sites and whose household members had symptoms in the past year were less likely to report unusual symptoms themselves. This could be due to the health seeking behavior of participants at this hospital site before further household transmission occurred. Although we could not prove causal effects, our data suggest that contact with animals and being scratched or bitten imposes a risk to human health and provides a pathway for zoonotic disease spillover among our study population [67]. A significantly higher proportion of PCR positive participants self-reported having unusual symptoms themselves or in other people they lived with in the past year compared to those with negative PCR tests. This suggests that the same population with sustained behaviors or practices are at a high risk to acquire infections, in general, and targeted preventative measures could be implemented for these at-risk populations.

Animal contact is prevalent and substantial among our study population with regard to livestock, poultry, and, to a smaller degree, wild animals. Our behavioral questionnaire, designed to gain insight into overall patterns around animal contact, indicated that contacts with animals occurred most frequently through handling and raising animals particularly poultry, swine and cattle, having animals come in the dwelling and slaughtering animals. This raises concern, since these animals that the participants had regular contact with carry several diseases—such as avian influenza, SARS [69], hantavirus, and rabies—that can be transmitted to humans and have epidemic or pandemic potential [70, 71]. Human infections of highly pathogenic avian influenza from poultry have been first reported in Thailand where poultry farming has been propagating in the last few decades [72]. Participants also reported handling and raising poultry and consuming raw or undercooked poultry, which are all risk factors of human avian influenza outbreaks [73, 74]. Consumption of pork, especially undercooked, and handling of swine as reported in our study pose a risk for Hepatitis E virus (HEV) as previous studies showed evidence of widespread HEV circulation in Thailand, and more importantly high HEV seroprevalence in swine farmers and those who consume pig organ meat [75–77]. It is of importance to note that contact with rodents and bats was also observed among our participants. One important observation from our data is that only 6 out of 29 bat guano miners interviewed in the study actually reported contact with bats. Therefore, bat and rodent contact are likely underestimated using survey instruments that rely on self-reporting. From our previous observations of this population, the majority of miners at this site had weekly direct or indirect contact with bats or bat feces. Bats are reservoirs of several zoonotic pathogens of global concern including severe acute respiratory syndrome (SARS) [28, 78], Nipah virus [79], Ebola [80], and rabies [81]. Rodents carry mammarenaviruses particularly lymphocytic choriomeningitis virus (LCMV) that have been found in Thailand [82] and are known to cause human illness [83] and hantaviruses that can occasionally be transmitted to humans [84]. Further examinations with serology together with behavioral data are needed to understand the prior exposure, animal-to-human transmission pathways and risk factors of viral spillover in this region.

### Zoonotic risk knowledge and perception of animal contact behaviors

Gender, age, and primary livelihood were shown to be statistically associated with animal contact (excluding dogs and cats contact), with older age groups having more frequent contact with animals than those under 40 years of age, and more males reporting contacts with animals than females. This might be due to the involvement of older male participants in different practices involving animal contact such as slaughtering, hunting, raising, and handling animals, putting them at higher risk of exposure, which may be an artifact of the significantly higher number of male participants with clinical symptoms at hospital sites. As our study showed that crop production workers with no more than a primary education, unemployed participants with no more than a primary education, and those aged between 40 and 59 were more likely to have contact with animals, future interventions for zoonotic risks reduction mitigation and educational activities in the communities should focus on these specific populations.

Even though participants under 20 years of age represented only 13% of our study population, 84% of our participants had a secondary education or less. This low educational attainment could be associated with participants’ limited knowledge about the disease risks associated with open wounds or in live animal markets [85–87]. Participants’ concern about disease outbreaks in live animal markets and knowledge about risk of open wounds were not found to differ between those who had contact with animals and those who did not. This could be due to the ubiquitous animal presence in these communities, lack of awareness of risk, and/or potentially to low risks perceptions [88–90]. This highlights the need for raising awareness among the older male population, those with low education attainment and bat guano miners about the zoonotic disease risks from indirect contact with animals. Risk mitigation education and activities are also most likely to be effective if focused on these specific population. There was also a lack of appropriate health seeking behavior among the high-risk groups with regular animal contact and those who lacked knowledge of risk of open wounds. Raising awareness of zoonotic diseases and educational messaging around treatment seeking behaviors will reduce the risk of infection from animals without completely discouraging their livelihood.

Given the prevalence of animal contacts among our study population, raising awareness of disease risks from contacts with poultry, livestock, and wildlife, having open wounds, and activities in a live animal market environment is critical. Gender appears to play a role in perception of risk, as more females recognized the possible disease outbreaks from live animal markets, which agrees with previous findings about zoonotic disease risk perception [91]. In addition, participants’ knowledge of risks influenced their health behavior, as among those who demonstrated knowledge of diseases from live animals and of open wounds, only 8% said they would keep working when bitten or scratched compared to 38% of those who lacked knowledge about risks who said they would keep working after they were bitten or scratched. This suggests that limited knowledge is still a barrier to health seeking behavior in these high-risk communities and makes early detection of zoonoses a challenge especially at the community level.

## Conclusion

Although this study does not establish causality between human–animal interactions and viral infection because we only had one-time measurements of exposure of interest (human–animal interactions) and the outcome (viral infection), the results from this study suggest that there is a very high interaction between humans and domestic and wild animals among surveyed communities in Thailand. A longitudinal study design, where we could record participants activities around animal-contact and conduct monthly molecular tests, would be needed to establish a true causality. Nonetheless, we highlight the most common pathways and livelihoods by which humans come into contacts with different animals in the area. Our findings on risk factors and risk perception also emphasize the need to improve public knowledge on zoonotic diseases and strengthen disease surveillance in the context of community practices. In the future, more targeted sampling in this region should include a wider, unbiased screening for potential zoonotic viruses and the use of multi-plex serology to identify evidence of past infection. Efforts to increase point-of-care detection of a diverse array of pathogens in hospitals and community settings are also needed, as well as studies to understand the behavioral risk factors associated with zoonotic transmissions. The biological–behavioral surveillance method used in this study will guide the targeted surveillance and behavioral interventions, contributing to the early-warning, detection, and prevention of emerging and re-emerging zoonotic diseases.

## Supplementary Information

**Additional file 1.** Standardized Human Questionnaire.

**Additional file 2. Fig. S1**. Type of interactions involving animals.** Fig. S2**. Type of animals that participants reported having contact with.

## Data Availability

All the PCR testing data are available in the PREDICT Emerging Pandemic Threats Project USAID Development Data Library (DDL) at https://data.usaid.gov/d/tqea-hwmr. Due to the confidentiality related to the protection of research participants, the full survey dataset will be available from the corresponding author upon reasonable request with certification of completed education in the protection of human subjects.

## Abbreviations

BP: Bootstrap support
COVID-19: Coronavirus disease 2019
HEV: Hepatitis E virus
LASSO: Least absolute shrinkage and selction operator
LCMV: Lymphocytic choriomeningitis virus
OR: Odds ratio
PCR: Polymerase chain reaction
RT-PCR: Reverse transcription-polymerase chain reaction
SARS: Severe acute respiratory syndrome
