# Supplementary material for: Behavioral–biological surveillance of emerging infectious diseases among a dynamic cohort in Thailand
Source: BMC Infect Dis. 2022 May 16;22:472. doi: 10.1186/s12879-022-07439-7 (PMC9109443; doi:10.1186/s12879-022-07439-7)
Supplement: Supplementary file 1 — Additional file 1. Standardized Human Questionnaire. [file 12879_2022_7439_MOESM1_ESM.pdf]

Human Questionnaire Form

Directions for completing the questionnaire

All questions are required unless otherwise indicated.

Directions for selecting modules for the interview

1. Select module(s) for site characterization
2. Add the Temporary Settlement Module if respondent dwelling is NOT permanent (Question 15)
3. Add all modules for work activities chosen in question 24 (Question 24)
4. Add Hunter Module if hunter/trapper/fisher is selected in Question 24 or "yes" to Question 52 "Since this time last year...".

Livelihood Module Table (based on response to Question 24)
Complete the module that corresponds with the livelihood chosen as follows:

extraction of minerals, gas, oil, timber	extractive industry module
crop production	crop production module
wildlife restaurant business	wildlife restaurant module
wild/exotic animal trade business	market and value chain module
rancher/farmer animal production business	animal production module
meat processing, slaughterhouse, abattoir	animal production module
zoo/sanctuary animal health care	zoos & sanctuaries module
hunter/trapper/fisher	hunter module
nurse, doctor, traditional healer, community health worker	hospital or clinic health professional module
If 'other' livelihood is livestock animal or product trade business	market and value chain module

example only do not use in field

If no additional modules are selected, the interview is complete.

Human Questionnaire Form ID Instructions

Enter the Site and Event Form ID barcode number to the grid located at the top of page 1 of the Human Questionnaire Form.

Enter the Human Questionnaire Form ID barcode number to grid located at the top of each associated module.

The barcode is located at the bottom right hand corner of each page of the Human Questionnaire section and the Site and Event Characterization Form.

Use the number after the dash (-) and fill the grid with the numbers from top to bottom.

Human Questionnaire Form

EIDITH
v1.4

Add Site and Event Form ID:

0	1	2	3	4	5	6	7	8	9
0	1	2	3	4	5	6	7	8	9
0	1	2	3	4	5	6	7	8	9
0	1	2	3	4	5	6	7	8	9
0	1	2	3	4	5	6	7	8	9
0	1	2	3	4	5	6	7	8	9

Site name and date: ____________________

(For reference only)

1. Consent Form Administered & Signed
   - ○ yes
   - ○ no

Participant ID: ______________

2. (Question removed)

3. Date of interview ____________________

4. Begin time of interview ____________
   (Example: 17:50)

5. End time of interview ____________
   (Example: 19:20)

6. Where are you conducting this interview?

   Village/Town/City ________________ Province/State ________________

   Latitude ____________________ Longitude ____________________

   Interviewer: Please collect GPS coordinates if administering using paper and pen.

7. Interviewer Observed Gender
   - ○ male
   - ○ female
   - ○ other

example only do not use in field

INTERVIEW/QUESTIONNAIRE BEGINS

Demographics Section

8. How old are you? ____________
   If the exact age is unknown, enter the respondent's estimated age.

9. Where do you live?

   Village/Town/City ________________ Province/State ________________

   Latitude ____________________ Longitude ____________________

   Interviewer: Probe for landmarks or nearest known site if area unknown.
   GPS coordinates to be identified and entered after completion of interview.

10. How long have you lived there? Select one option.
    - ○ <1 month
    - ○ 1 month - 1 year
    - ○ >1 - 5 years
    - ○ >5 - 10 years
    - ○ >10 years

11. How many other people live in the dwelling where you live? ______ (Not including participant)

    Skip to question 14 if answer is 0.

12. How many in the dwelling are children less than 5 years old? ______ (Not including participant)

13. How many in the dwelling are male? ______ (Not including participant)

316613-849572

Participant ID

____

(For reference only)

14. How many rooms are there in the dwelling where you live? ________
    (Do not include bathroom or kitchen)

15. Is the dwelling a permanent structure (that cannot be moved)?
    Interviewer: If answer is no, complete temporary settlement questionnaire.
    - ○ yes
    - ○ no

16. Do you get water from:
    Select all that apply.
    - ○ piped in water/water taps
    - ○ covered well
    - ○ uncovered well/pond/river
    - ○ water truck/rainwater harvest
    - ○ other: __________________

17. Do you treat your drinking water?
    - ○ yes
    - ○ no

18. **If yes,** how do you treat your water?
    Select all that apply.
    - ○ boil
    - ○ filter
    - ○ add chlorine or bleach
    - ○ solar disinfection
    - ○ other: __________________

19. Is your source for drinking water ever used by animals?
    - ○ yes
    - ○ no

20. In your dwelling is there a dedicated location for human solid waste/excreta?
    (Example: toilet, latrine, designated area)
    - ○ yes
    - ○ no

21. Do you have containers for storing food for the household?
    Select all that apply.
    - ○ yes, with covers
    - ○ yes, without covers
    - ○ no

example only do not use in field

Livelihood Section

In this section, I'd like to ask you about education and the kinds of work activities that you have done since this time last year.

22. What is the highest level of education you have completed?
    Select one option.
    - ○ primary school
    - ○ secondary school
    - ○ college/university/professional
    - ○ none

23. What is the highest level of education that your mother completed?
    Select one option. (Skip for Cameroon.)
    - ○ primary school
    - ○ secondary school
    - ○ college/university/professional
    - ○ none

316614-849572

Participant ID ____________________
(For reference only)

24. Since this time last year what are the activities you have done to earn your livelihood? Select all that apply
- ▢ extraction of minerals, gas, oil timber ***(extractive industry module)***
- ▢ crop production ***(crop production module)***
- ▢ wildlife restaurant business ***(wildlife restaurant module)***
- ▢ wild/exotic animal trade/market business ***(market and value chain module)***
- ▢ rancher/farmer animal production business ***(animal production module)***
- ▢ meat processing, slaughterhouse, abattoir ***(animal production module)***
- ▢ zoo/sanctuary animal health care ***(zoos & sanctuaries module)***
- ▢ protected area worker
- ▢ hunter/trapper/fisher ***(hunter module)***
- ▢ forager/gatherer/non-timber forest product collector
- ▢ migrant laborer
- ▢ nurse, doctor, traditional healer, community health worker ***(hospital or clinic health professional module)***
- ▢ construction
- ▢ other: ______________________________

If 'other' livelihood is livestock animal or product trade business ***(market and value chain module)***

25. If more than one activity was selected, what is the activity on which you spend the most time since this time last year? Select one option.
- ▢ extraction of minerals, gas, oil timber
- ▢ crop production
- ▢ wildlife restaurant business
- ▢ wild/exotic animal trade/market business
- ▢ rancher/farmer animal production business
- ▢ meat processing, slaughterhouse, abattoir
- ▢ zoo/sanctuary animal health care
- ▢ protected area worker
- ▢ hunter/trapper/fisher
- ▢ forager/gatherer/non-timber forest product collector
- ▢ migrant laborer
- ▢ nurse, doctor, traditional healer, community health worker
- ▢ construction
- ▢ other: ______________________________

26. Which best describes your job position? Select one option.
- ▢ manager/owner/foreman
- ▢ worker
- ▢ live and work at home independently (If chosen, skip to question 28)
- ▢ professional
- ▢ other: ____________________

27. Where do you work?

Village/Town/City ____________________ Province/State ____________________

Latitude ____________________ Longitude ____________________

Interviewer: Probe for landmarks or nearest known site if area unknown.
GPS coordinates to be identified and entered after completion of interview.

example only do not use in field

316615-849572

Medical History Section

Participant ID ____________ (For reference only)

In this section, I'm going to ask you about any illness or sickness that is not known or recognized in the community, including by medical or treatment providers.

28. Where do you usually get treatment for medical problems? Select all that apply.
    - ○ clinic/health center
    - ○ hospital
    - ○ mobile clinic
    - ○ community health worker
    - ○ traditional healer
    - ○ dispensary or pharmacy

29. Have you ever had an unusual illness with any of the following symptoms: Select all that apply. (READ ONLY SYMPTOMS)
    - ○ fever with headache and severe fatigue or weakness (encephalitis)
    - ○ fever with bleeding or bruising not related to injury (hemorrhagic fever)
    - ○ fever with cough and shortness of breath or difficulty breathing (SARI)
    - ○ fever with muscle aches, cough, or sore throat (ILI)
    - ○ fever with diarrhea or vomiting
    - ○ fever with rash
    - ○ persistent rash or sores on skin
    - ○ no (Skip to question 33)
    - ○ yes but, none of these symptoms-describe: ____________

30. Since this time last year, have you had any of these symptoms?
    - ○ yes
    - ○ no (Skip to question 33)

31. If yes, which ones? Select all that apply.
    - ○ fever with headache and severe fatigue or weakness (encephalitis)
    - ○ fever with bleeding or bruising not related to injury (hemorrhagic fever)
    - ○ fever with cough and shortness of breath or difficulty breathing (SARI)
    - ○ fever with muscle aches, cough, or sore throat (ILI)
    - ○ fever with diarrhea or vomiting
    - ○ fever with rash
    - ○ persistent rash or sores on skin
    - ○ yes but, none of these symptoms-describe: ____________

32. In your opinion, when you were sick, what caused this sickness? Select all that apply.
    - ○ contact with sick people
    - ○ contact with wild animals
    - ○ contact with other animals
    - ○ bad food or water
    - ○ bad spirits/witchcraft
    - ○ wound or injury
    - ○ I don't know
    - ○ other: ____________

33. Since this time last year, have any of the people you lived with had any of these symptoms?
    - ○ yes
    - ○ no (Skip to question 36)

34. If yes, which ones? Select all that apply.
    - ○ fever with headache and severe fatigue or weakness (encephalitis)
    - ○ fever with bleeding or bruising not related to injury (hemorrhagic fever)
    - ○ fever with cough and shortness of breath or difficulty breathing (SARI)
    - ○ fever with muscle aches, cough, or sore throat (ILI)
    - ○ fever with diarrhea or vomiting
    - ○ fever with rash
    - ○ persistent rash or sores on skin
    - ○ yes but, none of these symptoms-describe: ____________

35. Since this time last year, did anyone you lived with die from this illness?
    - ○ yes
    - ○ no

example only do not use in field

316616-849572

Human Questionnaire Form

Participant ID

____

(For reference only)

Movement Section

In this section, I'm going to ask you about any travel you have done since this time last year.

36. Have you traveled since this time last year?
If answer is no, skip to the next section.

- ◯ yes
- ◯ no

37. Where have you traveled since this time last year? Anywhere else?
Provide details, such as name of town, nearest (or most frequent) well known place if unknown by interviewer (to be linked to GPS coordinates later)
Collect up to 6 locations.

Interviewer: Probe for landmarks or nearest known site if area unknown. GPS coordinates to be identified and entered after completion of interview.

Village/Town/City ______ Province/State ______ Country ______

Latitude ______ Longitude ______

Notes: ______

Village/Town/City ______ Province/State ______ Country ______

Latitude ______ Longitude ______

Notes: ______

Village/Town/City ______ Province/State ______ Country ______

Latitude ______ Longitude ______

Notes: ______

Village/Town/City ______ Province/State ______ Country ______

Latitude ______ Longitude ______

Notes: ______

Village/Town/City ______ Province/State ______ Country ______

Latitude ______ Longitude ______

Notes: ______

Village/Town/City ______ Province/State ______ Country ______

Latitude ______ Longitude ______

Notes: ______

If there are more than six locations check here. ◯
Do not collect additional location information.

38. Why have you traveled?
Select all that apply.

- ◯ work
- ◯ visit family
- ◯ moved
- ◯ religious reasons
- ◯ holiday/vacation
- ◯ go to hospital/seek medical care
- ◯ go to market
- ◯ other: ______

example only do not use in field

316617-849572

Human Questionnaire Form

Animal Contact Section

Participant ID

____

(For reference only)

In this section, I'm going to ask you about the animals in your life.

If answered "no" under the "In your lifetime" column, then no answer is required under the "Since this time last year" column.

	In your lifetime...	Since this time last year...
39. Has an animal lived as a pet in or near your dwelling?	○ yes ○ no	○ yes ○ no
40. Have you handled live animals?	○ yes ○ no	○ yes ○ no
41. Have you raised live animals?	○ yes ○ no	○ yes ○ no
42. Have you shared a water source with animals for washing?	○ yes ○ no ○ don't know	○ yes ○ no ○ don't know
43. Have you seen animal feces in or near food before you have eaten it?	○ yes ○ no	○ yes ○ no
44. Have you eaten food after an animal has touched or damaged it? (Example: chew marks or scratches)	○ yes ○ no ○ don't know	○ yes ○ no ○ don't know
45. Do any animals come inside the dwelling where you live?	○ yes ○ no	○ yes ○ no
46. Have you cooked or handled meat, organs or blood from a recently killed animal?	○ yes ○ no	○ yes ○ no
47. Have you eaten raw or undercooked meat or organs or blood?	○ yes ○ no	○ yes ○ no
48. Have you eaten an animal that you knew was not well/sick?	○ yes ○ no ○ don't know	○ yes ○ no ○ don't know
49. Have you found a dead animal and collected it to eat or share?	○ yes ○ no	○ yes ○ no
50. Have you found a dead animal and collected it to sell it?	○ yes ○ no	○ yes ○ no
51. Have you been scratched or bitten by an animal?	○ yes ○ no	○ yes ○ no
52. Have you hunted or trapped an animal? (If answered "yes" to "Since this time last year" also administer the hunter module)	○ yes ○ no	○ yes ○ no
53. Have you slaughtered an animal?	○ yes ○ no	○ yes ○ no

example only do not use in field

316618-849572

Participant ID ____________________
(For reference only)

54. The last time you were scratched or bitten, or cut yourself while butchering or slaughtering, what did you do?
Select all that apply.

- ○ let someone else take over
- ○ wash wound with soap and water
- ○ rinse wound with water
- ○ bandage wound
- ○ visit doctor
- ○ nothing - kept working
- ○ never butcher or slaughter
- ○ n/a

55. Are there any risks associated with slaughtering or butchering when you have an open wound?
Interviewer: Do not read responses.

- ○ no
- ○ yes, but I don't know what they are
- ○ yes, it can make you sick
- ○ yes, it can poison you
- ○ yes, it can infect you with a disease
- ○ don't know
- ○ other: ________________________________

56. Interviewer: Circle all headings where “yes” was answered in the "Since this time last year" questions above.
Then ask which animals/mammals for each “yes” category.
Select all that apply.

	pet (39)	handled (40)	raised (41)	feces in or near food (43)	in house (45)	cooked/ handled (46)	eaten raw/ under cooked (47)	eaten sick (48)	found dead (49/50)	scratched/ bitten (51)	hunted/ trapped (52)	slaugh-tered (53)
rodents/shrews	○	○	○	○	○	○	○	○	○	○	○	○
bats	○	○	○	○	○	○	○	○	○	○	○	○
non-human primates	○	○	○	○	○	○	○	○	○	○	○	○
birds	○	○	○	○	○	○	○	○	○	○	○	○
carnivores	○	○	○	○	○	○	○	○	○	○	○	○
ungulates	○	○	○	○	○	○	○	○	○	○	○	○
pangolins	○	○	○	○	○	○	○	○	○	○	○	○
poultry/other fowl	○	○	○	○	○	○	○	○	○	○	○	○
goats/sheep	○	○	○	○	○	○	○	○	○	○	○	○
camels	○	○	○	○	○	○	○	○	○	○	○	○
swine	○	○	○	○	○	○	○	○	○	○	○	○
cattle/buffalo	○	○	○	○	○	○	○	○	○	○	○	○
dogs	○	○	○	○	○	○	○	○	○	○	○	○
cats	○	○	○	○	○	○	○	○	○	○	○	○

example only do not use in field

57. Are you worried about diseases or disease outbreaks in live animals in your local market?

- ○ yes
- ○ no

END OF MAIN QUESTIONNAIRE
