# Supplementary material for: Behavioral–biological surveillance of emerging infectious diseases among a dynamic cohort in Thailand
Source: BMC Infect Dis. 2022 May 16;22:472. doi: 10.1186/s12879-022-07439-7 (PMC9109443; doi:10.1186/s12879-022-07439-7)
Supplement: Supplementary file 2 — Additional file 2. Fig. S1. Type of interactions involving animals. Fig. S2. Type of animals that participants reported having contact with. [file 12879_2022_7439_MOESM2_ESM.docx]

**Additional File 1: Fig. S1: Type of interactions involving animals**

**Additional File 1: Fig. S2: Type of animals that participants reported having contact with**
